# Comparison of Generic Prescribing Patterns Among 340B-Eligible and Non-340B Prescribers in the Medicare Part D Program

**DOI:** 10.1001/jamahealthforum.2023.1026

**Published:** 2023-05-19

**Authors:** Sean Dickson, Katelyn James

**Affiliations:** 1 West Health Policy Center, Washington, DC

## Abstract

This cross-sectional study examines generic prescribing patterns for 2020 among 340B-eligible and non-340B clinicians in the Medicare Part D program to assess whether 340B revenue incentives may influence prescribing.

## Introduction

The 340B drug discount program allows certain federally designated health care entities (“covered entities”) to purchase retail and clinician-administered prescription drugs at a discounted rate, allowing them to generate revenue when reimbursed by insurers at standard, undiscounted rates.^1^ Covered entities include nonprofit hospitals and clinics that meet federal funding and patient characteristics criteria. The discount is greater on brand-name drugs than on generic drugs, generating concerns that 340B-eligible covered entities may overprescribe brand-name drugs to increase revenue.^2^ If true, this could increase patient cost sharing and result in higher overall prescription drug spending. Prior analysis found no difference in Medicare Part B drug spending across 340B and non-340B institutions,^3^ but to our knowledge, no analysis has been performed in the Medicare Part D market. In this cross-sectional study, we examine generic prescribing patterns for 2020 among 340B-eligible and non-340B clinicians in the Medicare Part D program to assess whether 340B revenue incentives may influence prescribing.

## Methods

We used an established method to identify whether a Medicare Part D prescription was written by a 340B-eligible covered entity.^4^ Briefly, we linked National Provider Identifiers in the Medicare Part D prescriber utilization file^5,6^ to the 340B covered entity database to determine whether a prescriber was 340B eligible based on the registered practice location. We matched drugs to US Pharmacopeia therapeutic class and summed generic and total prescriptions for 2020 within each class for 340B-eligible and non-340B prescribers. Given differences in therapeutic class mix across non-340B and 340B-eligible prescribers, we adjusted prescription count in each class for 340B prescribers to match the non-340B distribution while maintaining observed generic prescribing rates. We compared overall, adjusted overall, and class-level generic prescribing rates using χ^2^ tests, with *P* < .05 considered statistically significant. Per the decision guidance of the US Department of Health and Human Services, this cohort study was exempt from institutional review board approval and informed consent because it did not involve health care records and used only data that were publicly available. We followed the STROBE reporting guideline. Analyses were performed from October to December 2022.

## Results

Overall, 86.6% of 2020 Part D prescriptions were for generic drugs. For 340B-eligible prescribers, 86.4% of prescriptions were generic compared with 86.6% among non-340B prescribers (Figure). While statistically significant (odds ratio, 1.017; 95% CI, 1.016-1.018; *P* < .001), in practice, there was no apparent difference in generic prescribing by 340B status. After adjusting for differences in therapeutic class mix, 340B-eligible prescribers had an 86.6% generic prescribing rate, which was identical to the non-340B prescribers.

**Figure.**
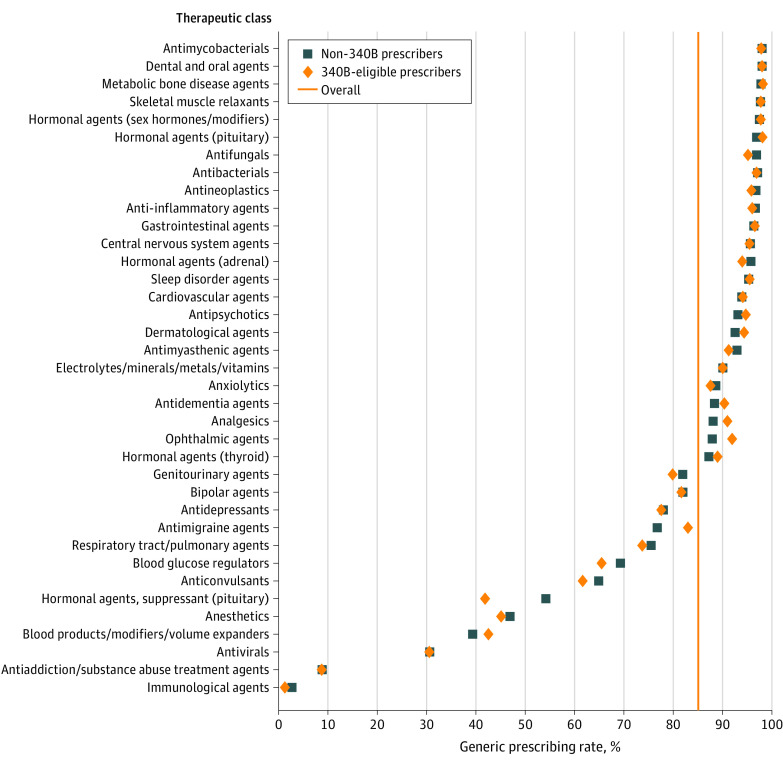
Generic Prescribing Rates in Medicare Part D by Therapeutic Class and by 340B-Eligible and Non-340B Prescribers, 2020

At the therapeutic class level, there were 11 classes where 340B-eligible prescribers had statistically significant higher generic prescribing rates and 17 classes where non-340B prescribers had statistically significant higher generic prescribing rates (Table). Of the categories with statistically significant differences, three-quarters had a difference less than 2 percentage points; antimigraine agents had the largest percentage-point difference, with 340B-eligible prescribers using generic formulations 84.6% of the time compared with 78.5% among non-340B prescribers. However, antimigraine agents only accounted for 1% of all prescriptions assessed; among cardiovascular agents, which accounted for a quarter of all prescriptions, 340B-eligible prescribers had higher generic rates (95.9%-95.7%).

**Table.** Generic Prescribing Rates by Therapeutic Class Among Non-340B and 340B Clinicians in Medicare Part D, 2020

Therapeutic class	Non-340B clinicians		340B clinicians		Difference in generic fill rate, pp	*P* value
	Generic fill rate, %	Total claims	Generic fill rate, %	Total claims		
Analgesics	90.1	44 337 590	92.7	2 827 952	2.6	<.001
Anesthetics	48.0	161 074	46.2	15 400	−1.8	<.001
Antiaddiction/substance abuse treatment agents	9.5	7 016 930	9.4	451 407	−0.1	<.001
Anti-inflammatory agents	98.6	113 0147	98.0	184 470	−0.6	<.001
Antibacterials	98.9	7 902 881	98.4	513 062	−0.4	<.001
Anticonvulsants	66.1	4 674 254	62.9	264 676	−3.3	<.001
Antidementia agents	90.2	12 427 456	92.0	791 893	1.9	<.001
Antidepressants	79.7	91 508 258	79.2	5 828 821	−0.5	<.001
Antifungals	99.1	419 863	96.7	15 474	−2.3	<.001
Antimigraine agents	78.5	7 391 723	84.6	637 260	6.0	<.001
Antimyasthenic agents	94.4	159 594	93.0	21 705	−1.4	<.001
Antimycobacterials	99.9	38 863	99.7	3866	−0.3	<.001
Antineoplastics	98.7	7 532 884	97.7	641 157	−1.0	<.001
Antipsychotics	95.0	9 449 604	96.4	576 418	1.4	<.001
Antivirals	31.8	3 155 982	31.4	355 736	−0.4	<.001
Anxiolytics	90.4	46 439 788	89.1	2 964 937	−1.2	<.001
Bipolar agents	83.6	14 055 406	83.4	814 664	−0.1	<.001
Blood glucose regulators	70.8	35 782 849	66.8	2 379 162	−4.0	<.001
Blood products/modifiers/volume expanders	40.2	22 028 432	43.3	1 804 239	3.1	<.001
Cardiovascular agents	95.7	177 478 905	95.9	13 223 421	0.1	<.001
Central nervous system agents	97.7	13 595 728	97.2	875 936	−0.4	<.001
Dental and oral agents	99.9	9 796 454	99.9	547 430	0.0	.84
Dermatological agents	94.6	10 773 872	96.2	663 590	1.5	<.001
Electrolytes/minerals/metals/vitamins	92.0	15 266 593	91.5	1 061 874	−0.5	<.001
Gastrointestinal agents	98.1	29 687 841	98.4	2 030 548	0.3	<.001
Genitourinary agents	83.7	26 263 776	81.4	1 948 024	−2.3	<.001
Hormonal agents (adrenal)	97.5	30 515 827	95.9	2 251 025	−1.5	<.001
Hormonal agents (sex hormones/modifiers)	99.4	1 675 767	99.5	106 502	0.0	.03
Hormonal agents (pituitary)	99.1	123 170	99.9	12 473	0.8	<.001
Hormonal agents (thyroid)	89.3	44 712 164	90.7	2 968 072	1.4	<.001
Hormonal agents, suppressant (pituitary)	55.3	40 030	42.9	3629	−12.3	<.001
Immunological agents	3.2	2 259 752	2.2	257 577	−1.1	<.001
Metabolic bone disease agents	99.8	7 698 951	99.8	520 664	0.0	<.001
Ophthalmic agents	89.6	19 122 563	93.9	2 264 544	4.3	<.001
Respiratory tract/pulmonary agents	77.0	45 709 070	75.2	3 158 286	−1.8	<.001
Skeletal muscle relaxants	99.5	4 310 272	99.6	282 270	0.1	<.001
Sleep disorder agents	97.4	7 455 120	97.4	410 357	−0.0	<.001
Overall	86.6	762 099 433	86.4	53 678 521	−0.2	<.001
Adjusted overall^a^	NA	NA	86.6	53 678 521	0.0	.55

Abbreviations: NA, not applicable; pp, percentage point.

^a^ The adjusted overall 340B-eligible generic prescribing rate adjusts the total claims in each therapeutic class to match the therapeutic class distribution among non-340B prescribers but maintains the observed generic prescribing rate. This adjustment accounts for differences in therapeutic class mix across 340B-eligible and non-340B prescribers.

## Discussion

In what is, to our knowledge, the first analysis of generic prescribing rates among 340B-eligible and non-340B Medicare Part D prescribers, we found no meaningful difference in overall generic prescribing rates. We observed variation in both directions at the therapeutic class level, but we found no evidence that 340B-eligible prescribers were systematically overprescribing brand-name drugs to generate revenue. This variation may result from differences in patient health status across 340B-eligible and non-340B prescribers, as 340B-eligible patients with Medicare are more likely to be dual eligible and/or disabled.^3^ Variation may also be more present in classes with lower overall utilization, as the highest-use class, cardiovascular agents, saw near-equal generic prescribing rates. Limitations of this study include possible errors in data matching and class-level aggregation that may have obscured prescribing variation for specific conditions. However, overall we found no evidence that the 340B program is leading to systematically higher spending on brand-name drugs in the Medicare Part D program.

## Notes

Supplement. Data Sharing Statement
