# Supplementary material for: Comparison of Generic Prescribing Patterns Among 340B-Eligible and Non-340B Prescribers in the Medicare Part D Program
Source: JAMA Health Forum. 2023 May 19;4(5):e231026. doi: 10.1001/jamahealthforum.2023.1026 (PMC10199350; doi:10.1001/jamahealthforum.2023.1026)
Supplement: Supplement. — Data Sharing Statement [file jamahealthforum-e231026-s001.pdf]

Data Sharing Statement

Dickson. Comparison of Generic Prescribing Patterns Among 340B-Eligible and Non-340B Prescribers in the Medicare Part D Program. *JAMA Health Forum*. Published May 19, 2023. doi:10.1001/jamahealthforum.2023.1026

Data
Data available: No

Additional Information
Explanation for why data not available: All data is publicly available
